# Supplementary material for: Comparison of the efficacy among different interventions for radiodermatitis: A Bayesian network meta‑analysis of randomized controlled trials
Source: PLoS One. 2024 Apr 10;19(4):e0298209. doi: 10.1371/journal.pone.0298209 (PMC11006171; doi:10.1371/journal.pone.0298209)
Supplement: S1 Appendix — (DOCX) [file pone.0298209.s002.docx]

**S1 Appendix. Search Strategy.**

**1. PubMed search strategy（n=260）**

Mesh:Radiodermatitis

Entry Terms:

Radiodermatitides

Radiation-Induced Dermatitis

Radiation Induced Dermatitis

Dermatitis, Radiation-Induced

Dermatitides, Radiation-Induced

Dermatitis, Radiation Induced

Radiation-Induced Dermatitides

Radiation Recall Dermatitis

Dermatitides, Radiation Recall

Dermatitis, Radiation Recall

Radiation Recall Dermatitides

Radiation Recall Reaction

Radiation Recall Reactions

Reaction, Radiation Recall

Reactions, Radiation Recall

Recall Reaction, Radiation

Recall Reactions, Radiation

randomized controlled trial [Publication Type] OR randomized [Title/Abstract] OR placebo[Title/Abstract]

Combined Search strategy =(("Radiodermatitis"[Mesh]) OR ((((((((((((((((((Radiodermatitis[Title/Abstract]) OR (Radiodermatitides[Title/Abstract])) OR (Radiation-Induced Dermatitis[Title/Abstract])) OR (Radiation Induced Dermatitis[Title/Abstract])) OR (Dermatitis, Radiation-Induced[Title/Abstract])) OR (Dermatitides, Radiation-Induced[Title/Abstract])) OR (Dermatitis, Radiation Induced[Title/Abstract])) OR (Radiation-Induced Dermatitides[Title/Abstract])) OR (Radiation Recall Dermatitis[Title/Abstract])) OR (Dermatitides, Radiation Recall[Title/Abstract])) OR (Dermatitis, Radiation Recall[Title/Abstract])) OR (Radiation Recall Dermatitides[Title/Abstract])) OR (Radiation Recall Reaction[Title/Abstract])) OR (Radiation Recall Reactions[Title/Abstract])) OR (Reaction, Radiation Recall[Title/Abstract])) OR (Reactions, Radiation Recall[Title/Abstract])) OR (Recall Reaction, Radiation[Title/Abstract])) OR (Recall Reactions, Radiation[Title/Abstract]))) AND (randomized controlled trial[Publication Type] OR randomized[Title/Abstract] OR placebo[Title/Abstract])

n=260

**2. Embase Search Strategy（n=311）**

Emtree:radiation dermatitis

Entry Terms：

Radiodermatitides

Radiation-Induced Dermatitis

Radiation Induced Dermatitis

Dermatitis, Radiation-Induced

Dermatitides, Radiation-Induced

Dermatitis, Radiation Induced

Radiation-Induced Dermatitides

Radiation Recall Dermatitis

Dermatitides, Radiation Recall

Dermatitis, Radiation Recall

Radiation Recall Dermatitides

Radiation Recall Reaction

Radiation Recall Reactions

Reaction, Radiation Recall

Reactions, Radiation Recall

Recall Reaction, Radiation

Recall Reactions, Radiation

Details：

#5 #3 AND #4 311

#4 'placebo':ab,ti OR 'random':ab,ti OR 'double-blind':ab,ti 833144

#3 #1 OR #2 14329

#2 'radiodermatitides':ab,ti OR 'radiation-induced dermatitis':ab,ti OR 'radiation induced dermatitis':ab,ti OR 'dermatitis, radiation-induced':ab,ti OR 'dermatitides, radiation-induced':ab,ti OR 'dermatitis, radiation induced':ab,ti OR 'radiation-induced dermatitides':ab,ti OR 'radiation recall dermatitis':ab,ti OR 'dermatitis, radiation recall':ab,ti OR 'dermatitides, radiation recall':ab,ti OR 'radiation recall dermatitides':ab,ti OR 'radiation recall reaction':ab,ti OR 'radiation recall reactions':ab,ti OR 'reaction, radiation recall':ab,ti OR 'reactions, radiation recall':ab,ti OR 'recall reaction, radiation':ab,ti OR 'recall reactions, radiation':ab,ti 462

#1 radiation AND dermatitis 14298

**3. Cochrane Search Strategy（n=490，trial=488）**

Mesh:Radiodermatitis

Entry Terms:

Radiodermatitides

Radiation-Induced Dermatitis

Radiation Induced Dermatitis

Dermatitis, Radiation-Induced

Dermatitides, Radiation-Induced

Dermatitis, Radiation Induced

Radiation-Induced Dermatitides

Radiation Recall Dermatitis

Dermatitides, Radiation Recall

Dermatitis, Radiation Recall

Radiation Recall Dermatitides

Radiation Recall Reaction

Radiation Recall Reactions

Reaction, Radiation Recall

Reactions, Radiation Recall

Recall Reaction, Radiation

Recall Reactions, Radiation

Details:

#1 MeSH descriptor: [Radiodermatitis] explode all trees 257

#2 (Radiodermatitides):ti,ab,kw or (Radiation-Induced Dermatitis):ti,ab,kw or (Radiation Induced Dermatitis):ti,ab,kw or (Dermatitis, Radiation-Induced):ti,ab,kw or (Dermatitides, Radiation-Induced):ti,ab,kw or (Dermatitis, Radiation Induced):ti,ab,kw or (Radiation-Induced Dermatitides):ti,ab,kw or (Radiation Recall Dermatitis):ti,ab,kw or (Dermatitis, Radiation Recall):ti,ab,kw or (Dermatitides, Radiation Recall):ti,ab,kw or (Radiation Recall Dermatitides):ti,ab,kw or (Radiation Recall Reaction):ti,ab,kw or (Radiation Recall Reactions):ti,ab,kw or (Reaction, Radiation Recall):ti,ab,kw or (Reactions, Radiation Recall):ti,ab,kw or (Recall Reaction, Radiation):ti,ab,kw or (Recall Reactions, Radiation):ti,ab,kw 300

#3 #1 OR #2 490

Trials 488

**4. Web of science Search Strategy（n=666）all databases**

Mesh:Radiodermatitis

Entry Terms:

Radiodermatitides

Radiation-Induced Dermatitis

Radiation Induced Dermatitis

Dermatitis, Radiation-Induced

Dermatitides, Radiation-Induced

Dermatitis, Radiation Induced

Radiation-Induced Dermatitides

Radiation Recall Dermatitis

Dermatitides, Radiation Recall

Dermatitis, Radiation Recall

Radiation Recall Dermatitides

Radiation Recall Reaction

Radiation Recall Reactions

Reaction, Radiation Recall

Reactions, Radiation Recall

Recall Reaction, Radiation

Recall Reactions, Radiation

Details:

#1 TS=(Radiodermatitis OR radiodermatitises OR Radiation-Induced Dermatitis OR Radiation Induced Dermatitis OR Dermatitis, Radiation-Induced OR Dermatitides, Radiation-Induced OR Dermatitis, Radiation Induced OR Radiation-Induced Dermatitides OR Radiation Recall Dermatitis OR Dermatitides, Radiation Recall OR Dermatitis, Radiation Recall OR Radiation Recall Dermatitides OR Radiation Recall Reaction OR Radiation Recall Reactions OR Reaction, Radiation Recall OR Reactions, Radiation Recall OR Recall Reaction, Radiation OR Recall Reactions, Radiation) 6197

#2 TS=(randomized controlled trial OR randomized OR placebo OR random OR double-blind) 3,147,948

#1 AND #2 666
