# Supplementary material for: Comparison of the efficacy among different interventions for radiodermatitis: A Bayesian network meta‑analysis of randomized controlled trials
Source: PLoS One. 2024 Apr 10;19(4):e0298209. doi: 10.1371/journal.pone.0298209 (PMC11006171; doi:10.1371/journal.pone.0298209)
Supplement: S1 File — (DOCX) [file pone.0298209.s003.docx]

| **Corticosteroid**  Difluprednate (MYSER ointment 0.05%)  Mometasone  0.1% mometasone furoate  Betamethasone0.1% |
| --- |
| **PBMT**  MLS^®^ laser therapy (LT)  Light emitting diode (LED) photomodulation  Photobiomodulation therapy  Red light phototherapy (RLPT) |
| **Oral agents**  Oral glutamine  L- Glutamine |
| **Barrier films and dressings**  Mepitelfilm (Mepitel film; Mepilex Lite)  Xonrid^®^  Hydrofilm  StrataXRT^®^  KeraStat (KeraStat(R) Cream; Keratin) |
| **Natural and traditional herbs**  Curcumin (Nanocurcumin; Curcumin C3 complex; Curcumin cream; Vicco^®^ turmeric cream [VTC])  Henna (Alpha ointment)  Chamomile (Chamomile gel)  Calendula (Calendula cream) |
| **Miscellaneous agents**  Trolamine (Trolamine cream)  Hyaluronicacid (Hyaluronic acid; HÀ formulation) |

**File 3 BOX.** Included interventions in the Network Meta-analysis by therapeutic class.
